# Immunomodulatory role of spleen tyrosine kinase in chronic inflammatory and autoimmune diseases

**DOI:** 10.1002/iid3.934

**Published:** 2023-07-27

**Authors:** Yaqi Zhou, Yaowen Zhang, Wei Yu, Yufen Qin, Heng He, Fengxian Dai, Yibo Wang, Fengqin Zhu, Guangxi Zhou

**Affiliations:** ^1^ Department of Clinical Medicine Jining Medical University Jining Shandong China; ^2^ Department of Gastroenterology Affiliated Hospital of Jining Medical University, Jining Medical University Jining Shandong China

**Keywords:** autoimmune disease, chronic inflammation, signaling pathway, spleen tyrosine kinase

## Abstract

**Background:**

The high prevalence of chronic inflammatory diseases or autoimmune reactions is a major source of concern and affects the quality of life of patients. Chronic inflammatory or autoimmune diseases are associated with many diseases in humans, including asthma, rheumatoid arthritis, systemic lupus erythematosus, inflammatory bowel disease and cancer. Splenic tyrosine kinase (SYK) is a non–receptor tyrosine kinase that plays an important role in immune receptor signalling in immune and inflammatory responses.

**Methods:**

This is a review article in which we searched for keywords “splenic tyrosine kinase”, “inflammation” and “autoimmune diseases” in published literature such as Pubmed and Web of Science to collect relevant information and then conducted a study focusing on the latest findings on the involvement of SYK in chronic inflammatory or autoimmune diseases.

**Results:**

This paper reviews the regulation of Fcγ, NF–κB, B cell and T cell–related signalling pathways by SYK, which contributes to disease progression in chronic inflammatory and autoimmune diseases such as airway fibrosis, inflammatory skin disease and inflammatory bowel disease.

**Conclusion:**

This paper shows that SYK plays an important role in chronic inflammatory and autoimmune diseases. syk targets hematological, autoimmune and other inflammatory diseases and therefore, inhibition of SYK expression or blocking its related pathways may provide new ideas for clinical prevention and treatment of inflammatory or autoimmune diseases.

## Graphical abstract

This review summarizes the mechanisms of SYK‐mediated signal transduction and highlights recent findings on the role of SYK in chronic inflammatory or autoimmune diseases.

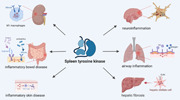

**Abbreviations**
AAA: the abdominal aortic aneurysms
AD: atopic dermatitis
BCRs: B‐cell receptors
BLNK: B cell linker protein
CARD9: caspase recruitment domain‐containing protein 9
CLEC‐2: the C‐type lectin‐like receptor‐2
CLL: chronic lymphocytic leukemia
ERK: the extracellular signal‐regulated kinase
FcγRI: Fc receptor for human immunoglobulin G1
FcγRs: Fcγ receptors
FLN: Filamin
HCC: hepatocellular carcinoma
HSC: hepatic stellate cells
ITAM: immunoreceptor tyrosine‐based activation motif
NO: nitric oxide
PI3K: phosphoinositide 3‐kinase
PLCγ: phospholipase Cγ
SH2: Src homology 2
SLE: systemic lupus erythematosus
SYK: spleen tyrosine kinase
TLR4: Toll‐like receptor‐4
ZAP70: zeta‐chain associated protein kinase 70

## 1 INTRODUCTION

Spleen tyrosine kinase (SYK) plays a key role in immune receptor signaling during the immune or inflammatory response. SYK is expressed on T lymphocytes, B lymphocytes, NK cells, macrophages, dendritic cells (DCs), mast cells and intestinal epithelial cells. It is found on both hematopoietic and nonhematopoietic cells. SYK works as an intermediate in intracellular signal transduction, transmitting activation signals from membrane receptors downstream.^1^ Activated immune cells are recruited to the site of injury during the pathogenesis of inflammatory or autoimmune diseases, accompanied by the regulated production of variable pro‐ or anti‐inflammatory mediators. This helps to eliminate invading pathogens and maintain tissue homeostasis.^2^ However, when inflammatory factors persist, inflammation becomes chronic, immune tolerance is destroyed, and an imbalance in immune cell numbers in diseased tissues leads to immune system disorders, which can further lead to autoimmunity. Immune cell receptors, including Fcγ receptors (FcγRs), B‐cell receptors (BCRs), and NK cell receptors, activate SYK through a series of chemical reactions, which then have a vital role in chronic inflammation or autoimmune diseases. We deepened our study of pathways upstream of SYK that exert positive proinflammatory effects in this review. At the same time, we demonstrate that SYK‐targeted therapy can effectively treat circulatory system, blood system, other inflammatory and autoimmune diseases. We believe that our study can help researchers fully understand SYK, which can help develop therapeutic strategies against inflammation, immunity and other related diseases by targeting or modulating SYK.

## 2 STRUCTURE OF SYK

**Figure 1:**
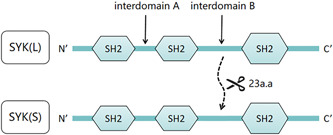
Structure of spleen tyrosine kinase. Spleen tyrosine kinase (SYK) has two tandemly repeated Sγc homology 2 (SH2) structural domains and a C‐terminal kinase structural domain connected by interdomain linkers A and B. SYK encodes two transcripts, including the full‐length SYK(L) and the interspliced SYK(S). The SYK(S) subtype is deficient in a 69 bp sequence, resulting in 23 residues missing from interdomain B.

SYK is a 72 kDa nonreceptor tyrosine kinase with two tandemly repeated Src homology 2 (SH2) structural domains and one C‐terminal kinase structural domain joined by interdomain linkers A and B^3^, ^4^ (Figure 1). In the steady state, the SH2 domain and the C‐terminal kinase domain have intermolecular interactions and the two N‐terminal SH2 domains are distorted, thus preventing them from binding to the immunoreceptor tyrosine‐based activation motif (ITAM), so that SYK remains in an inactive conformation.^5^ However, SYK can be activated by the Src family via phosphorylation of ITAM adaptors. ITAMs are found in the cytoplasmic domain of immune receptors including FcγRs, BCR, and NK cell receptors.^6^ Src family kinases phosphorylate ITAM motifs, and the SH2 domain binds to phosphorylated tyrosine residues in ITAM. In other words, SH2‐N of SYK is recruited to the C‐terminal pYxxL of ITAM, while SH2‐C is recruited to the N‐terminal pYxxL of ITAM.^4^ In addition, there are receptors such as the C‐type lectin‐like receptor‐2 (CLEC‐2) and the C‐type lectin‐like receptor NKp65, which contains only a YXX (L/I) motif in its cytoplasmic region and is known as hemITAM.^7^

SYK encodes full‐length SYK (L) and an alternatively spliced SYK (S) (Figure 1).^8^ Overexpression of labeled SYK subtypes in H1155 cells revealed that SYK (L) is mainly located in the nucleus and cytoplasm, whereas SYK (S) is only distributed in the cyto‐plasm. The interaktion of SYK(L) with the universally expressed transcriptional regulator YinYang 1 inhibits epithelial‐mesenchymal transformation by inactivating the transcription of SNAI2 (encoding the transcription factor SLUG) transcription. SYK (L) is critical in the development, metastasis and treatment of tumors.^9^

SYK has an activation state distinct from phosphorylated ITAM, which activates kinases through autophosphorylation of the interdomain junction tyrosine.^10^ The binding of the SH2 domain to the intracellular Toll‐IL‐1 receptor domain of Toll‐like receptor‐4 (TLR4) can also result in conformational change in SYK, LPS‐induced TLR4 activation, and subsequent SYK‐mediated cascades in proinflammatory signaling amplification. Drugs that target SYK and TLR4 have the potential to be developed as novel and innovative treatment options for Helicobacter pylori‐associated gastropathy.^11^ Very low‐density lipoprotein is rich in apolipoprotein C3 protein, which binds to TLR2/4. It further induces the downstream adaptor protein SCIMP to upregulate and bind Lyn, which directly phosphorylates and activates SYK. Activated SYK mediates TRPM2‐dependent calcium influx and subsequent generation of reactive oxygen species, which activate NLRP3 inflammasomes and promote atherosclerosis and myocardial infarction.^12^

The pathogenic role of SYK gene variants or related fusion proteins in inflammatory and autoimmune diseases is of wide interest. Lin et al. identified SYK Ser 550 mutations in six patients with multiorgan inflammation and immune dysregulation leading to SYK hyperactivation. In a mouse SYK p.Ser544Tyr mutant model corresponding to human SYK p.Ser550Tyr, histological examination of the mice's tails revealed an increase in immune cells and signs of arthritis. There were large aggregates of macrophages, osteoclasts and T lymphocytes in the ankle joint of the mice. After treatment with R406, the mice also showed a remission of the inflammation described above.^13^ TEL is a transcriptional repressor that has been found to fuse with tyrosine kinases and is closely associated with many hematological tumors. Some researchers transferred the TEL‐SYK fusion gene into fetal mouse hepatocytes and found increased levels of STAT5 phosphorylation in the hepatocytes, but failed to inhibit STAT5 phosphorylation after treatment with a JAK inhibitor, suggesting that TEL‐SYK controls STAT5 signaling independent of JAK2. Meanwhile, they found that TEL‐SYK caused bone marrow fibrosis and abnormal proliferation in mice.^14^ In addition, the ITK‐SYK fusion protein has been shown to promote T‐cell lymphoma and inflammation.^15^

## 3 SYK SIGNALING PATHWAY AND INFLAMMATION

Several intermediary molecules are involved in the transmission of signal downstream of SYK, including members of the Vav family, the phospholipase Cγ (PLCγ) subtype, the regulatory subunit of phosphoinositide 3‐kinase (PI3K) and members of the SH2 domain‐containing leukocyte protein family and B cell linker protein (BLNK). These molecules may form proximal receptor signaling complexes to activate multiple pathways such as the Vav Guanine Nucleotide Exchange Factor 1 VAV1/Rac, IkB kinase/nuclear factor kappa B (NF‐κB) signaling, Ca2+ and protein kinase C signaling, RAS homologous family, reactive oxygen species production and phagocytosis, and PI3K/AKT signaling process. They may also be involved in transcriptional regulation through the extracellular signal‐regulated kinase (ERK) and T nuclear factor pathways, which are activated by Ca^2+^.^16^, ^17^ MyD88 also plays a key role in SYK signal transduction. Gurung et al.^18^ studied inflammatory skin diseases in mice caused by mutations in the Ptpn6 gene. They found that SYK phosphorylates tyrosine residues 180 and 278 on MyD88. The SHP1 encoded by Ptpn6 binds and inhibits the activation of SYK, thereby suppressing the phosphorylation of MyD88.^18^ Yi et al.^3^ studied the macrophage inflammatory response. The data suggest that MyD88 interacts with SYK through tyrosine 58 residues in its ITAM to activate the MyD88‐SYK axis and the downstream NF‐kB signaling pathway. Phosphorylation of the hemITAM motif of the platelet receptor CLEC‐2 induces phosphorylation of the SYK Y342 site and regulates the hemITAM‐mediated signaling pathway.^7^ The mechanism of SYK activation by NKp65 is different from that of CLEC‐2. SYK is not directly recruited to hemITAM, but perhaps through phosphorylation of Src‐family kinase members.^19^

### 3.1 SYK and Fcγ signaling pathway

FcγRs are receptors for the C‐terminus of immunoglobulin Fc regions. Immuno‐globulin binding to antigens renders the Fc region allosterically accessible to bind to Fc receptors on the cell membrane. The γ chain is a common subunit that carries ITAM, which initiates receptor assemblage and signaling by sequentially activating Src and SYK. The receptor assembly and signal transduction pathways have various biological effects.

In addition, Lopez‐Sanz et al.^20^ observed overexpressed FcγRs in the adventitial and medial layers of human and mouse abdominal aortic aneurysms (AAA), the deteriorative weakening and dilation of the abdominal aorta. It is known as a chronic inflammation that involves innate and adaptive immunity. Fc receptor was inactivated in gamma‐knockout mice, and SYK phosphorylation was reduced, whereas it was elevated in AAA lesions in WT mice. This suggests that the subsequent vascular activity of FcγR is dependent on SYK phosphorylation. FcγR could be an interesting therapeutic targeting molecule to control vascular immune inflammatory injury in people suffering from AAA.^20^

**Figure 2:**
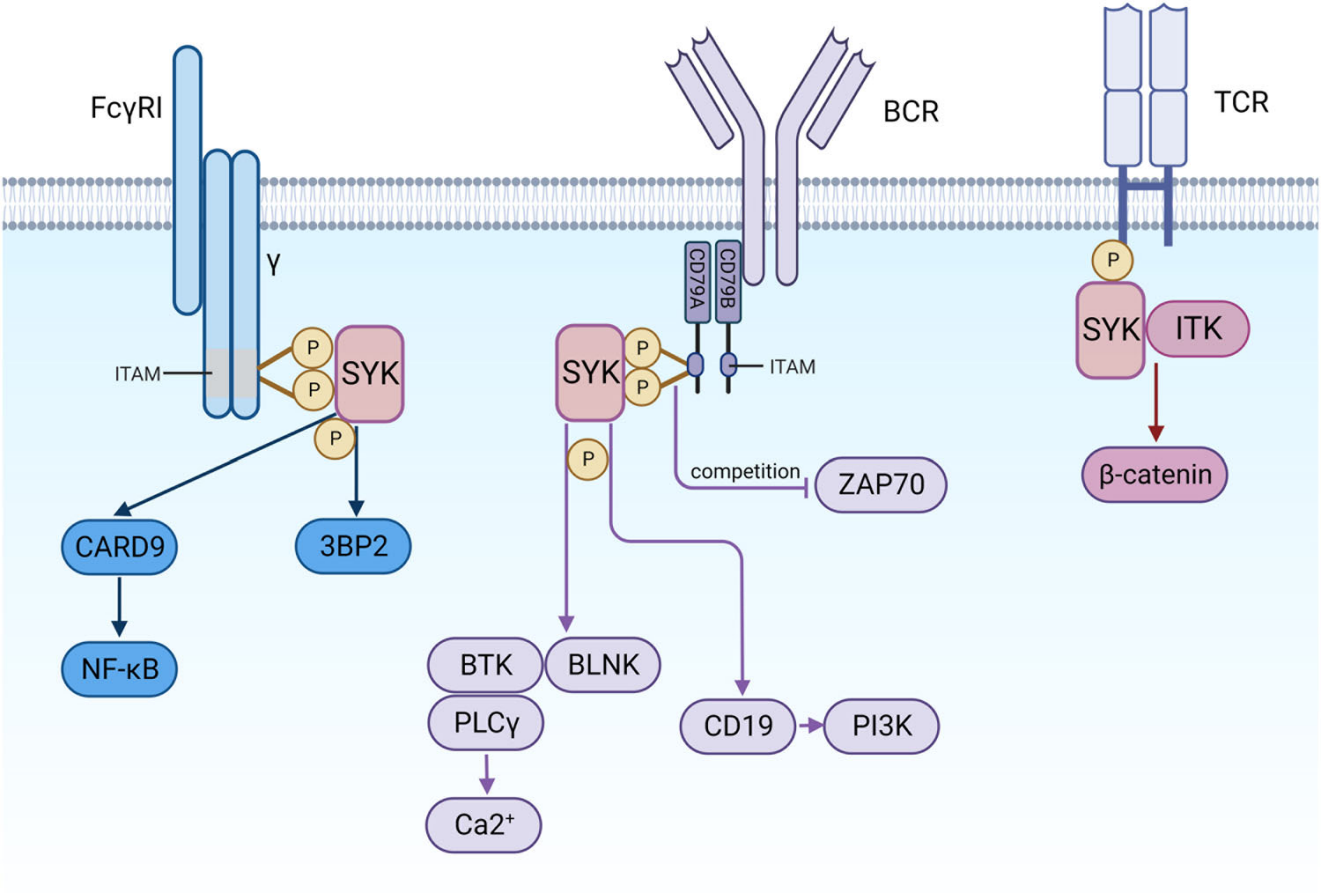
SYK mediated multiple signaling pathways. Fcγ receptor (FcγR) is activated by immunoglobulin and its γ chain carries ITAM for receptor assembly and signal transduction by activating SYK. SYK phosphorylates adaptor protein c‐Abl SH3 domain‐binding protein‐2 in response to FcγRI cross‐linking. CARD9 couples SYK activation to the NF‐κB pathway. SYK attaches the ITAM structural domains of CD79A and B, leading to their activation. SYK activates BCR signaling to activate two distinct downstream pathways, namely Ca2+ signaling via BLNK, BTK, and PLCγ and PI3K signaling via CD19 phosphorylation. The phospholipid phosphatidylinositol‐4, 5‐diphosphate is activated by PI3K phosphorylation and converted to phosphatidylinositol‐3, 4, 5‐triphosphate, then recruiting several important downstream signaling molecules and therapeutic targets. β‐Catenin is a cytoskeletal protein mainly involved in cell adhesion. Activation of the T cell receptor (TCR) activates ITK phosphorylation. The ITK‐SYK fusion gene can phosphorylate β‐catenin. ITAM, immunoreceptor tyrosine‐based activation motif; NF‐κB, nuclear factor kappa B; SYK, spleen tyrosine kinase.

SYK phosphorylates the adaptor protein c‐Abl SH3 domain‐binding protein‐2 (3BP2) in response to Fc receptor for human immunoglobulin G1 (FcγRI) cross‐linking. Chihara et al.^17^ found that SYK‐dependent tyrosine phosphorylation of 3BP2 was required for FcγR‐mediated chemokine expression and phagocytosis in IFN‐γ‐stimulated U937 cells (Figure 2). Furthermore, many immune cells express FcγRs. The release of neutrophil extracellular traps (NETs) can protect against inflammation.^21^ SYK is involved in the formation of Fcγ‐mediated NETs and is essential for neutrophil function during phagocytic activity.^22^

In addition to immune cells, FcγR regulates platelet function. Filamins (FLNs) consists of three actin‐filament‐crosslinking homologous genes, FLNa, FLNb, and FLNc. FLNa interacts with SYK after platelet activation, and its mutations cause filamentous disease, in which SYK activation is inhibited due to defects in the SYK‐FLNA association.^23^ The destruction of platelets by spleen macrophages is dependent on FcγR. Therefore, targeting SYK to block the FcγR signaling may be useful in treating thrombocytopenia‐related diseases.^24^

### 3.2 SYK and NF‐κB pathway

The SYK/NF‐κB signaling pathway is associated with cancer. Caspase recruitment domain protein 9 (CARD9) is dependent on the regulation of SYK, which in turn initiates NF‐κB transcriptional activization and the production of cytokines and chemokines (Figure 2). During fungal infection in mice, some investigators found that the SYK‐CARD9 signaling pathway is activated as a downstream effector of CLR and induces NF‐κB signal transduction. The SYK–CARD9 signaling axis recognizes symbiotic intestinal fungi, which has a protective effect against inflammation‐related cancers.^25^

**Figure 3:**
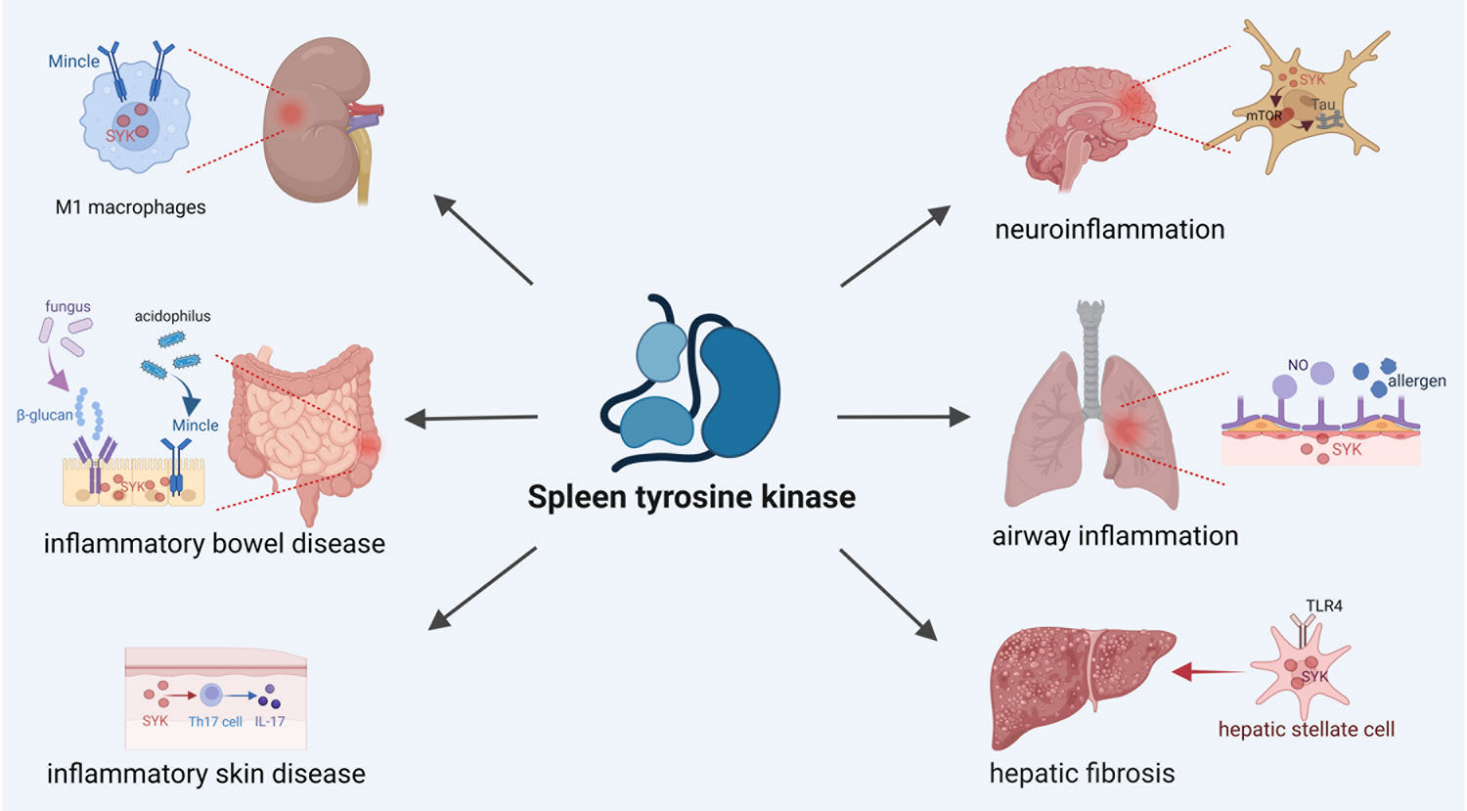
The role of SYK in chronic inflammatory and autoimmune diseases. Allergens and nitric oxide stimulate airway epithelial cells to phosphorylate SYK and exacerbate asthma or chronic obstructive pulmonary disease. SYK serves to promote liver fibrosis by interacting with Toll‐like receptor‐4 (TLR4) on hepatic stellate cells. IL‐17A production by skin Th17 cells is closely associated with psoriasis and atopic dermatitis, and SYK promotes IL‐17A secretion by Th17 cells. β‐glucan is one of the components of fungal cell walls that induces SYK phosphorylation through receptors expressed on intestinal epithelial cells. Mincle is a receptor expressed by Lactobacillus acidophilus on intestinal epithelial cells that may also promote SYK phosphorylation and be involved in the pathogenesis of colitis. SYK is a mammalian target of rapamycin (mTOR)‐related regulator, and activation of SYK leads to increased phosphorylation and accumulation of Tau. M1 macrophages are key cells involved in kidney injury and Mincle is a key factor in maintaining the M1 phenotype, which can be involved in kidney inflammation through activation of SYK. SYK, spleen tyrosine kinase.

We found new insights into the role of NF‐κB in renal inflammation. M1 macrophages are critical cells involved in renal obstruction and are a key component of renal injury. Inflammatory M1 macrophage phenotypes are enhanced and sustained by the Mincle‐SYK pathway (Figure 3). Quercetin and isoliquiritigenin, are both flavonoids that block the conversion of macrophages from M1 to M2 and prevent the development of a fibrotic phenotype during the conversion process. Mincle is a crucial factor in the maintenance of the M1 macrophage phenotype through a SYK‐dependent mechanism involving TLR4/NF‐κB signal transduction. In models of acute kidney injury and unilateral ureteral obstruction, treatment with isoliquiritigenin, quercetin and the SYK inhibitor BAY61‐3606 all downregulated Mincle protein levels in renal macrophages, blocked the Mincle/SYK/NF‐κB signal transduction and significantly reduced renal inflammation and fibrosis.^26^, ^27^

### 3.3 SYK and B cell receptor signaling pathway

SYK is required for proper B cell growth and function. BCR binding triggers signal transduction through the Src family of kinases, resulting in phosphorylation of CD79A and CD79B. SYK recruits to the ITAM structural domains on CD79A and B and activates them.^28^ SYK activates BCR signaling for activation of two distinct downstream signal transduction pathways: phosphoinositide 3‐kinase signaling via CD19 and PI3K adapter protein phosphorylation and Ca2+ signaling via BLNK, BTK, and PLCγ. Activated PI3K‐δ phosphorylates and transforms phosphatidylinositol 4,5‐bisphosphate into phosphatidylinositol 3,4,5‐trisphosphate, recruiting several important downstream signaling molecules and therapeutic targets (Figure 2).^29^ Therefore, the lack of SYK expression can prevent B cells from differentiation and maturation.^30^ Wang et al.^13^ found that in B‐cell malignancies, over‐activated SYK binds to several antiapoptotic factors, which suggests that SYK may function as a tumor promoter. SYK promotes survival in B‐cell carcinoma through chronic signaling of the BCR pathway. Active SYK is expressed in acute or chronic lymphocytic leukemia (CLL) and in subgroups of EBV‐associated B‐cell lymphoma. Knockdown or inhibition of SYK can cause apoptosis.^25^ Munshi et al. demonstrated that mutant MyD88 activates SYK, a major driver of growth and survival of MyD88‐mutated lymphoma cells. The combination of ibrutinib and SYK inhibitors is synergistically lethal to MyD88 mutant B cells, which provides new ideas for the clinical treatment of B‐cell lymphoma.^31^

T cells express a tyrosine kinase structurally similar to SYK, zeta chain‐associated protein kinase 70 (ZAP70), which acts similarly to SYK in the transduction of signals to T cell receptors (TCRs). Recently, Sadras et al.^29^ have found that aberrant expression of ZAP70 is a common characteristic of several B‐cell malignancies. In B cells, aberrantly expressed ZAP70 is competitive with SYK in BCR signaling, leading to a shift in SYK from negative selection to complementary PI3K signal transduction and enhancing B cell survival. The co‐expression of SYK and ZAP70 significantly accelerates B‐CLL development in vivo. Moreover, co‐expression of SYK and ZAP70 significantly accelerated the development of B‐CLL in vivo. In addition, SYK and ZAP70 kinases were co‐expressed with other B‐cell malignancies besides CLL. The current value of SYK as an immunotherapeutic target requires further research into more effective and specialized inhibitors.^29^

Increased SYK phosphorylation after BCR cross‐linking was found in peripheral blood B cells of patients with active systemic lupus erythematosus (SLE). Pohlmeyer et al.^32^ reported that the selective SYK inhibitor lanraplenib reduced the deposition of immunoglobulin G in the glomeruli. Mice treated with lanraplenib showed reduced concentrations of serum proinflammatory cytokines. Thus, lanlaprinib may be effective in the prevention of disease worsening with lupus nephritis and SLE by inhibiting B‐cell maturation in patients.^32^

### 3.4 SYK and T cell related signaling pathways

T cells differentiate into different cell subtypes, such as CD8+ T cells, CD4+ T cells, Treg cells and memory T cells, which regulate and control the immune response. β‐Catenin is a cytoskeletal protein mainly involved in cell adhesion.^33^ Activation of the TCR activates ITK phosphorylation, which in turn regulates T cell differentiation and function. The ITK‐SYK fusion gene can phosphorylate the Wnt/β‐catenin signal transduction pathway in effector T cells or Tregs, and activation of Wnt/β‐catenin can promote colon inflammation and colon cancer (Figure 2). The researchers established an adjuvant‐induced arthritis mouse model and found that intraperitoneal injection of the tyrosine kinase inhibitor AG126 reduced the number of CD8+ T cells and increased the number of Treg cells in the knee joints of the mice, significantly reducing the severity of arthritis.^34^

### 3.5 SYK and other signaling pathways

SYK plays a role in autophagy and foam cell formation via the MEK/ERK pathway. Das et al.^35^ found that SYK activates the MEK/ERK pathway and triggers a cascade of responses induced by interacting with the novel thrombin‐induced platelet protein STLT1 and CD64. STLT1 interacts with FcγRI on the surface of macrophages and activates the downstream MAP kinase signaling cascade via SYK, which in turn activates NF‐κB. Activated NF‐κB leads to the secretion of tumor necrosis factor‐α by macrophages, which is an essential component in limiting the progression of chronic inflammation. This finding may lead to developing new treatment strategies for atherosclerosis.^35^

## 4 THE ROLE OF SYK IN OTHER INFLAMMATORY AND IMMUNE DISORDERS

SYK acts as an important mediator of the immune response in inflammatory and autoimmune diseases. There are many SYK‐targeted inhibitors in experimental and clinical use, and this kinase has become a prominent target for the development of therapeutic strategies. SYK signaling pathway plays a key role in DCs and neutrophils during sepsis‐induced acute kidney injury. SYK inhibitor R406 reduces inflammatory cytokines and oxidative stress in DCs and neutrophils.^36^ We will also focus on the critical role of SYK in airway inflammation, fibrosis, inflammatory bowel disease (IBD), inflammatory skin disease and neuroinflammation.

### 4.1 SYK and airway inflammation

SYK acts as a key component in inducing allergic airway inflammation and hyperresponsiveness. Airway inflammation caused by allergens increases SYK expression in the lung. Inflammation and reactivity in the airways are associated with elevated levels of sphingosine 1‐phosphate. Sphingosine 1‐phosphate and angiotensin‐II induce SYK phosphorylation in smooth muscle cells. Nitric oxide (NO) is a bronchoconstriction regulator (Figure 3). SYK has been associated with NO production in airway epithelial cells, but Tabeling et al.^37^ found that SYK promotes airway contraction in an NO‐independent manner, partly by activating RhoK and p38 MAPK. Inhibition of SYK can reduce inflammation in allergic airways, hyperreactivity, and lung collagen deposition. SYK inhibitors, such as BI 1002494 and BAY 61‐3606, may offer promising treatments for chronic and acute asthma attacks. In addition, Li et al.^38^ synthesized Eapp‐2, a 3‐arylbenzofuran derivative with potent anti‐inflammatory and SYK‐inhibitory properties. Eapp‐2 markedly inhibited the phosphorylation of SYK and modulated the downstream NF‐κB or NLRP3 pathways. In asthmatics and chronic obstructive pulmonary disease patients, SYK may be an effective therapeutic target. Acute lung injury is a severe inflammatory condition of the lung caused by a dysregulated inflammatory response. In a mouse model of lipopolysaccharide‐induced acute lung injury, inhibition of SYK signaling using R406 reduces oxidative stress, airway inflammation, and myeloperoxidase activity in immune cells.^39^ This suggests that R406 is a potential treatment for acute lung injury.

### 4.2 SYK and fibrosis

The development of liver fibrosis and fibrosis‐associated hepatocellular carcinoma (HCC) are both caused by chronic inflammation. Hepatic stellate cells (HSCs) are an important component of liver fibrosis. SYK(L) is mainly an expression in non‐neoplastic liver tissues and is significantly downregulated in HCC. Instead, SYK(S) is oncogenic and promotes the intrusion and metastasis of HCC cells. In particular, SYK(L) is essential for liver fibrosis in vitro. SYK promotes liver fibrosis by activating HSC. The expression levels of these two SYK isoforms could be potential biomarkers for the monitoring of HCC development. SGS‐9973, PRT062607, R406, and BAY‐61‐3606 are all SYK inhibitors that have significant in vitro anti‐fibrotic activity.^8^ Interestingly, Torres‐Hernandez et al.^40^ reported that inhibiting SYK is dependent on TLR4 for its anti‐fibrotic effects (Figure 3). Inhibition of SYK by piceatannol and PRT062607 inhibits HSC activation after TLR4 ligation, reverses T cell depletion and promotes the expression of protective factors by CD4+ T cells in liver fibrosis. PRT062607 completely inhibits SYK signals and is considered more selective.^40^

Alhazmi et al.^10^ showed that mutations in cystic fibrosis transmembrane conductance regulators cause a genetic disorder that leads to defective ciliary clearance and mucus accumulation in the lung. As a result, patients are highly susceptible to infection by Pseudomonas aeruginosa. The cystic fibrosis transmembrane conductance regulator is phosphorylated by SYK and its expression at the plasma membrane is reduced. The SYK inhibitor piceatannol can inhibit P. aeruginosa infection. R406 significantly inhibited the secretion of proinflammatory markers from human macrophages and lung epithelial cells infected with P. aeruginosa, thereby attenuating the development of pulmonary cystic fibrosis.^10^

### 4.3 SYK and IBD

IBD is an autoimmune disease. The cause of IBD is still unknown, but many studies have implicated that SYK may have a role in the pathogenesis of IBD. Gong et al.^41^ alleviated colitis in a mouse model of DSS‐induced colitis after administration of Piceatannol. Meanwhile, the researchers additionally used a nanoparticle encapsulating piceatannol, which provided high local concentrations of the drug and reduced drug degradation, thereby promoting prolonged pharmacological activity and efficacy.^42^ The results showed that this nanoparticle also improved intestinal flora dysbiosis.^41^ Macrophages are key gatekeepers of intestinal immune homeostasis, and intestinal macrophages derived from circulating monocytes have an important impact on the development of IBD. Biagioli et al.^43^ found that SYK is activated by DAP12 and induces M1 phenotypic differentiation. DAP12 is a homodimer containing ITAMs. DAP12 activates SYK phosphorylation and initiates inflammatory signaling in IBD cascade, leading to differentiation of intestinal macrophages towards the M1 phenotype and mediating inflammation‐induced immune dysfunction in experimental colitis.^43^ It has been shown that Mincle senses the release of spliceosome‐associated protein 130 from damaged intestinal epithelial cells, which activates SYK.^44^ Lactobacillus acidophilus is one of the major bacterial genera found in the mammalian gut and Mincle is a surface receptor for Lactobacillus acidophilus. Some researchers have found that Mincle interacts with the S‐layer on the surface of Lactobacillus acidophilus in a Ca^2+^‐dependent manner (Figure 3). In addition, CARD9 is a downstream molecule of Mincle and SYK induces pro‐ and anti‐inflammatory cytokine production via CARD9 in DCs.^45^ Gut fungi have a significant role in human intestinal homeostasis and one of the components of the fungal cell wall is β‐glucan. The glucan receptor Dectin‐1 is expressed in intestinal epithelial cells and recognizes many pathogenic fungi. β‐glucan induces SYK phosphorylation and Dectin‐1 is dependent on SYK transduction signals (Figure 3). SYK inhibition reduced the secretion of β‐glucan‐induced chemokines (e.g., IL‐8 and CCL2) from the intestinal epithelium. The above suggests a role for intestinal epithelial cells in fungal‐induced IBD and experimental colitis and other intestinal inflammatory conditions.^46^

### 4.4 SYK and inflammatory skin diseases

Inflammatory skin diseases are inflammatory disorders caused by allergies, autoimmune diseases, ulcers, etc. Cutaneous DCs secrete IL‐6 and IL‐23, which stimulate the polarization of TH0 cells towards Th17 cells. Psoriasis is a common chronic inflammatory skin condition caused by abnormalities in the immune system, and IL‐17A produced by skin Th17 cells and keratinocytes is closely linked to the pathogenesis of psoriasis (Figure 3). Under the induction of IL‐17A in skin keratinocytes, SYK can interact with Act1‐TRAF6 to induce the production of CCL20, which depends on the SYK‐mediated NF‐κ B signaling pathway.^47^ R406 inhibits TH17 cells by altering the signaling cascade response of DCs. The researchers used the TLR7 agonist imiquimod in mice to induce psoriasis inflammation. R406 treatment can significantly inhibit the expression of Th17 in CD11c+dc of mouse dorsal skin.^48^ Therefore, inhibition of SYK may be a treatment for psoriasis. In addition, UVB induces SYK phosphorylation in skin keratinocytes by activating reactive oxygen species and EGFR. R406 ameliorates mouse skin damage by inhibiting UV‐induced production of ROS, inflammasomes and inflammatory factors.^49^

Atopic dermatitis (AD) is the most common chronic recurring inflammatory skin condition associated with the overactivation of mast cells. Cryptotanshinone is a lipid‐soluble compound derived from salvia miltiorrhiza. Cryptotanshinone inhibits SYK phosphorylation and downstream target molecules such as PLCγ, IKKβ, and PKC, thereby inhibiting mast cell degranulation.^50^ ASN002 is the first oral dual JAK/SYK inhibitor for AD patients that significantly inhibits TH2 and TH17‐related pathways involved in the pathogenesis of AD and improves the epidermal barrier.^51^ In a randomized, double‐blind, placebo‐controlled Phase 1b study in 36 patients with moderate to severe AD, a higher proportion of patients (83.3%; *p* = .03) achieved greater than 50% improvement in lesion area and severity after receiving ASN002 80 mg daily compared to placebo (22.2%). Patients receiving ASN002 80 mg (−4.7 ± 2.1, *p* = .01) had a smaller but not statistically significant reduction in pruritus scores compared to the placebo group (−1.6 ± 1.8). The results show that ASN002 has a good therapeutic effect.^52^ In a double‐blind, controlled Phase 1B study in 11 patients with cutaneous lupus erythematosus treated with topical GSK2646264 or placebo for 28 days, investigators found that GSK2646264 was well tolerated and no new safety concerns were identified.^53^ Using proteomic and transcriptomic approaches, Gudjonsson et al.^54^ found that the inflammatory response in septic sweat gland inflammation is associated with SYK pathway activation. In summary, inhibition of SYK may be a therapeutic strategy for inflammatory skin diseases.

### 4.5 SYK and neuroinflammation

As mentioned above, SYK is important in inflammatory and adaptive immune responses and thus may be involved in neuroinflammation observed in neuro‐degenerative diseases. SYK is a key regulator of the rapamycin signaling pathway in mammals, and activation of SYK leads to increased phosphorylation and accumulation of Tau (Figure 3).^55^ AG126 is a key inhibitor of the development of neuroinflammatory disorders. Autism spectrum disorder is a neurological disorder with a neuroinflammatory response. AG126 acts as a neuroprotective and reduces neuroimmune dysfunction in a mouse model of autism treatment.^56^, ^57^ Some researchers found that inhibition or knockdown of SYK reduced activation of the mammalian rapamycin signaling pathway and increased tau degradation without affecting tau production, which is of great significance in case of neurodegenerative diseases.

## 5 CONCLUSION

Inflammation is an important protective mechanism against harmful stimuli, and if left untreated, it can have long‐term chronic effects. This paper reviews the role of SYK in the regulation of pathways such as Fcγ and NF‐κB from recent literature and investigates the role of SYK in chronic inflammatory and autoimmune diseases. In addition, this work suggests that inhibiting SYK expression or blocking its related pathways may provide new ideas for the clinical prevention and treatment of inflammatory or autoimmune diseases.

## AUTHOR CONTRIBUTIONS

**Yaqi Zhou**: conceptualization; visualization; writing—original draft. **Yaowen Zhang**: investigation; visualization. **Wei Yu**: investigation; visualization. **Yufen Qin**: investigation. **Heng He**: resources. **Fengxian Dai**: resources. **Yibo Wang**: resources. **Fengqin Zhu**: funding acquisition; project administration; writing—review & editing. **Guangxi Zhou**: funding acquisition; project administration; writing—review & editing.

## CONFLICT OF INTEREST STATEMENT

The authors declare no conflicts of interest.

## Data Availability

Not applicable.
